# Inhibition of c-Fos expression attenuates IgE-mediated mast cell activation and allergic inflammation by counteracting an inhibitory AP1/Egr1/IL-4 axis

**DOI:** 10.1186/s12967-021-02932-0

**Published:** 2021-06-15

**Authors:** Hui-Na Wang, Kunmei Ji, Li-Na Zhang, Chu-Chu Xie, Wei-Yong Li, Zhen-Fu Zhao, Jia-Jie Chen

**Affiliations:** Department of Biochemistry and Molecular Biology, School of Basic Medical Sciences, Laboratory Department of South China Hospital, Health Science Center, Shenzhen University, No. 1066 Xueyuan Road, Nanshan District, Shenzhen, 518060 People’s Republic of China

**Keywords:** Mast cells, FcεRI signaling, c-Fos, Egr1, T-5224

## Abstract

**Background:**

Activator protein-1 (AP1), a c-Fos–JUN transcription factor complex, mediates many cytobiological processes. c-Fos has been implicated in immunoglobulin (Ig)E activation of mast cells (MCs) via high-affinity IgE Fc receptor (FcεRI) binding. This study examined c-Fos involvement in MC activation and tested the effects of the c-Fos/AP1 inhibitor T-5224 on MCs activation and allergic responses.

**Methods:**

In vitro studies were conducted with two MC model systems: rat basophilic leukemia cells (RBLs) and mouse bone marrow derived mast cells (BMMCs). MC degranulation and effector functions were examined with β-hexosaminidase release and cytokine secretion assays. c-Fos/AP1 was inhibited with T-5224. c-Fos activity was suppressed with short hairpin RNA targeting c-Fos (shFos). In vivo immune responses were evaluated in passive cutaneous anaphylaxis (PCA) and ovalbumin-induced active systemic anaphylaxis (ASA) models, as well as in an oxazolone (OXA)-induced model of atopic dermatitis, a common allergic disease.

**Results:**

c-Fos expression was elevated transcriptionally and translationally in IgE-stimulated MCs. c-Fos binding of the *Egr1* (early growth response 1) promoter upregulated *Egr1* transcription, leading to production of interleukin (IL)4. T-5224 reduced FcεRI-mediated MC degranulation (evidenced by β-hexosaminidase activity and histamine levels) and diminished EGR1 and IL4 expression. T-5224 attenuated IgE-mediated allergic responses in PCA and ASA models, and it suppressed MC-mediated atopic dermatitis in mice.

**Conclusion:**

IgE binding can activate MCs via a c-Fos/Egr1/IL-4 axis. T-5224 suppresses MC activation in vitro and in vivo and thus represents a promising potential strategy for targeting MC activation to treat allergic diseases.

**Supplementary Information:**

The online version contains supplementary material available at 10.1186/s12967-021-02932-0.

## Introduction

Allergic diseases are disruptive to quality of life, can cause serious morbidity, and their incidence has been on the rise [1]. Mast cells (MCs) play a key role in immunoglobulin (Ig)E mediated allergic reactions [2]. Binding of high-affinity IgE Fc receptors (FcεRIs) on the surface of MCs by antigen (Ag)-linked IgE antibodies activates the IgE/FcεRI pathway, which promotes cellular degranulation and the release of cytoinflammatory factors [3, 4]. FcɛRI aggregation induces phosphorylation of a linker molecule [5] and a signaling cascade involving Bruton’s tyrosine kinase, phospholipase γ, and protein kinase C [6], leading to activation of downstream signaling pathways—including calcium signaling [7, 8], mitogen-activated protein kinase (MAPK) [9] and nuclear factor κB [10] pathways—which initiate MC degranulation and cytokine production.

Elucidating molecular mechanisms that modulate MC activation could provide crucial insights into the pathophysiology of allergic diseases. Recently, FcεRI-stimulated MC activation and anaphylaxis were shown to be dampened by antagonism of an orphan nuclear receptor called nuclear receptor subfamily 4 group A member 1, which acts in opposition to the anti-inflammatory liver kinase B1/adenosine monophosphate-activated protein kinase axis [11]. In addition, FcεRI-mediated MC activation has been shown to be negatively regulated by regulator of calcineurin 1 [12].

Activator protein-1 (AP-1)—a transcription factor complex formed by dimerization of c-Fos, a leucine zipper protein, with c-Jun—modulates signaling pathways involved in many cytobiological processes, including proliferation, differentiation, transformation, and cell death [13]. AP-1–dependent regulation of cytokine transcription requires the presence of c-Fos [14], and IgE/Ag treatments upregulate c-Fos transcription in MCs [15, 16]. In c-Fos-deficient MCs, FcɛRI-mediated degranulation can be inhibited by down-regulation of degranulation genes [13, 17]. Experiments examining anaphylactic processes have shown that released cytokines can induce AP-1-dependent MC secretion of interleukin (IL)6 consequent to activation of the *IL6* gene promoter [18]. The molecular mechanism by which FcεRI activation leads to upregulation of c-Fos expression has not been clarified. Moreover, clinical translation studies of the effects of c-Fos/AP-1 inhibition on IgE/Ag-activated MCs and allergic responses are needed.

Here, we used next-generation RNA sequencing (RNA-Seq) and real time quantitative reverse transcriptase (qRT)-polymerase chain reaction (PCR) to analyze the MC transcriptional response to IgE/Ag activation. We identified MC activation-associated proteins that interact with c-Fos, including early growth response (EGR), IL, and chemokine (C–C motif) ligand (CCL) proteins [19, 20]. We were particularly interested in the potential involvement of EGR1 because it regulates IL-4 secretion in FcεRI-activated MCs [21]. We conducted qRT-PCR and western blotting to measure *Egr1* expression levels in stimulated MCs, with and without anti-Fos silencing short hairpin RNA (ShFos). To explore the intracellular mechanism of c-Fos involvement in MC activation, we examined the effects of the c-Fos/AP-1 inhibitor T-5224 on inflammatory cytokine expression and MAPK signaling. Finally, we examined T-5224 effects on MC activation responses in in vivo models.

## Materials and methods

### Reagents

T-5224 (PubChem CID: 23626877) was purchased from TargetMol (Shanghai, China). Monoclonal DNP-specific IgE, DNP-HSA, and 4-nitrophenyl *N*-acetyl-β-d-glucosaminide were obtained from Sigma-Aldrich (St. Louis, MO). Aluminum adjuvant was purchased from Thermo Scientific (Waltham, MA). Evans blue, ketotifen fumarate (PubChem CID: 5282408), formamide and toluidine blue were purchased from Meilun Biotechnology Co., Ltd (Dalian, China).

### Animals

Female Balb/c mouse pups were used in accordance with ethical protocols. Female Balb/c mice (6–8 weeks, 18–20 g) from Guangdong Medical Laboratory Animal Center (Benxi, Liaoning, China) were bred in an aseptic-specified pathogen-free environment with controlled humidity (55 ± 10%) and temperature (24 ± 1 °C). Animal experiments were carried out according to protocols approved by our University’s Animal Care and Use Committee and in compliance with the Guidelines on Animal Welfare of the School of Medicine of Shenzhen University. Mouse health was monitored twice daily, and early humane euthanasia was conducted in cases of weight loss > 15% body weight, lethargy, inability to stand, or anorexia.

### Cell culture

Rat basophilic leukemia cells (RBLs; RBL-2H3 cell line from Cellcook Biotechnology, Guangzhou, China) were cultured in complete Dulbecco’s modified Eagle medium with 4.0 mM l-glutamine, sodium pyruvate, 100 U/mL penicillin, 100 µg/mL streptomycin, non-essential amino acid (Solarbio, Beijing, China) and 10% fetal bovine serum (Gibco, Grand Island, NY) in a humidified incubator at 37 °C, 5% CO_2_.

### Preparation of BMMCs

BMMCs were isolated from 6 to 8-week-old female Balb/c mouse femurs. The animals were euthanized with CO_2_ and submerged in 70% ethanol; then intact femurs were removed. Sterile endotoxin-free medium was flushed through bone shafts repeatedly with a syringe and needle. A suspension of collected bone marrow cells was centrifuged (300×*g*, 5 min). The spun-down cells were cultured (0.5 × 10^6^ nucleated cells/mL) in RPMI 1640 media supplemented with 10% fetal bovine serum, penicillin (100 U/mL), streptomycin (100 μg/mL), sodium pyruvate (10 mM), l-glutamine (2 mM), 4-(2-hydroxyethyl)-piperazine ethane sulfonic acid (10 mM), and recombinant stem cell factor and IL-3 (10 ng/mL each). After ~ 5 weeks in culture, BMMC purity was ~ 95%, as determined by flow cytometry of cell-surface CD117 and FcεRI [22].

### Cytotoxicity

Cell viability was evaluated with a Cell Counting Kit 8 assay kit (MedChem Express, Monmouth Junction, NJ). RBLs (2 × 10^3^/well) and BMMCs (1 × 10^4^/well) were each cultured in 96-well plates and incubated with T-5224 for 24 h. Kit-provided solution was added wells and incubated at 37 °C for 1 h. Absorbance at 450 nm was detected by a multi-well plate reader (Bio-Rad, Hercules, CA). Viability was calculated as relative absorbance expressed as a percentage of control values.

### β-Hexosaminidase release

β-Hexosaminidase release served as a MC degranulation index [23]. Dinitrophenol (DNP)-targeting IgE (50 ng/mL)-sensitized RBLs or BMMCs were pretreated with T-5224 for 1 h. After two phosphate buffered saline (PBS) washes, cells were stimulated with DNP-HSA (100 ng/mL) for 30 min at 37 °C. Supernatant aliquots (50 µL) were transferred to 96-well plates and incubated with an equal volume of substrate solution (1 mM 4-nitrophenyl-*N*-acetyl-β-d-glucosaminide) for 90 min at 37 °C. To obtain total β-hexosaminidase release, cells in control wells were lysed with 0.1% Triton X-100 before plate reading. The reaction was terminated with 150 μL of stop solution (0.1 M Na_2_CO_3_ and NaHCO_3_). Absorbance at 405 nm was detected by a multi-well plate reader (Bio-Rad, Hercules, CA).

### RNA-Seq

RNA-seq was performed with a BGISEQ-500 sequencer (Beijing Genomics Institute, Beijing, China) [24]. Total RNA was extracted from RBLs with Trizol. The sequencing data were filtered with SOAPnuke (v1.5.2) [25] by removing reads that contained the sequencing adapter or had a low-quality (< 5) base ratio > 20%. The remaining clean reads were stored in FASTQ format and mapped to the reference genome using HISAT2 (v2.0.4) [26]. The reads were aligned in RSEM (v.1.2.12) with Bowtie (v2.2.5) [27]. Differential expression was analyzed with DESeq2 (v1.4.5) [28], and gene expression heatmaps were drawn in pheatmap (v1.0.8). Our RNA-seq data were deposited in GEO (accession GSE161317).

### Real-time qRT-PCR

Total cellular RNA was isolated with an RNeasy Mini Kit (Qiagen, Duesseldorf, Germany) and reverse transcribed into cDNAs with a TIAN Script II RT Kit (Tiangen Biotech, Beijing, China) and primers (Additional file 1: Table S1) according to the manufacturer’s instructions. Real-time qRT-PCRs were performed with TB Green® Premix ExTaq™ (Takara, Tokyo, Japan) in a qTOWER 2.2 system (Analytik Jena, Upland, CA). Expression levels were computed by normalizing relative to *GAPDH* (glyceraldehyde 3-phosphate dehydrogenase) mRNA levels with the 2^−ΔΔCt^ technique. The primers were shown in Additional file 1: Table S1.

### Western blotting

Cell lysate preparation and immunoblotting were performed as previously described [29]. Anti-DNP IgE (50 ng/mL)-sensitized RBLs (5 × 10^5^/well in 6-well plates) were pretreated with T-5224 for 1 h and then stimulated with DNP-HSA for 4 h (p–c-Fos FRA1, and EGR1) or 30 min (others). The cells were washed with PBS twice and lysed with 200 µL RIPA buffer containing protease inhibitor cocktail (MedChem Express, Monmouth Junction, NJ). Lysed samples were cooled on ice for 15 min and centrifuged at 12,000 rpm for 10 min at 4 °C. Protein concentrations were measured with a BCA kit (Beyotime, Beijing, China). Equal amounts of lysate were separated by 10% sodium dodecyl sulfate–polyacrylamide-gel electrophoresis and transferred to polyvinylidene difluoride membranes.

Membranes were incubated with primary antibodies at 4 °C, and then incubated with horse radish peroxidase-conjugated anti-rabbit antibody for 1 h at room temperature. The following rabbit antibodies were purchased from Cell Signaling Technology (Danvers, MA) and applied at 1:1000 dilutions: p-p44/42 MAPK (ERK1/2) (#4370, Thr202/Thr204, monoclonal); p44/42 MAPK (ERK1/2) (#4695, monoclonal); JNK (**#**9252, polyclonal); p-JNK (Thr183/Tyr185) (#4668, monoclonal); p38 (**#**8690, monoclonal); p-p38 (#4511, monoclonal). The following rabbit monoclonal antibodies were purchased from Abcam (Cambridge, MA, USA): c-Fos (ab134122, 1:2000), FosB (ab184938, 1:10,000) and EGR1 (ab133695, 1:2000). Mouse anti-GAPDH monoclonal antibody (sc-25778) was purchased from Santa Cruz Biotechnology (Santa Cruz, CA) (1:1000). Second antibodies included anti-rabbit (#7074, 1:5000) and anti-mouse (#7076, 1:5000) IgG-horseradish peroxidase (HRP) were both from Cell Signaling Technologies., Inc. Protein bands were visualized using the enhanced chemiluminescence (Meilun, Dalian, China) and analyzed using ImageJ software (ImageJ 1.80v; National Institutes of Health, Bethesda, MA, USA).

### Chromatin immunoprecipitation (ChIP)

RBLs were collected as previously described [30]. Proteins were crosslinked to DNA by adding 270 µL of 37% formaldehyde to 10 mL culture medium (final concentration, 1%). After 10-min incubations at 37 °C, reactions were quenched by adding 0.125 M glycine for 5 min at room temperature. After washing with ice-cold PBS twice, fixed cells were lysed with 1 mL RIPA buffer (MedChem Express, Monmouth Junction, NJ) on ice for 10 min. After centrifugation, nuclei were sonicated (30% amplitude, 20 sets of 15-s bursts) for four rounds. Debris was removed by centrifugation at 13,000 rpm at 4 °C for 10 min; supernatants were transferred to new tubes. Lysates were precleared with protein A/G beads before being incubated with c-Fos antibodies (Cell Signaling Technology, Danvers, MA) or IgG (Santa Cruz, CA) at 4 °C overnight with rotation. The immunocomplex was washed (once each) in low salt, high salt, and LiCl solutions and washed twice in TE buffer. Bead-bound immunocomplexes were eluted with 200 µL elution buffer. To reverse histone-DNA crosslinks, samples were combined with 5 M NaCl and heated at 65 °C for 4 h. DNA product was purified in a spin column system (Omega Bio-Tek, Norcross, GA).

### Knockdown of c-Fos

We cloned shFos (5′-GCAGACCGAGATTGCCAATTT-3′) and *GFP* mRNA as a negative control (shNC, 5′-GCAAGCTGACCCTGAAGTTCAT-3′) into pLKO.1-puro vectors (Sigma, St. Louis, MO). Sequences were confirmed by DNA sequencing (Beijing Genomics Institute, Shenzhen, China). Liposomes (Bioinno-Profei Tech, Qingdao, China) were applied to enable cell transfection.

### Dual luciferase promoter–reporter assay

Transient transfection was conducted with liposome transfection reagent. RBLs were seeded in 24-well plates and transfected with the AP-1 luciferase-reporter plasmid pAP1-Luc (Promega, USA), and compared to control plasmid pGL3-basic or pGL3-control. Activities of Firefly luciferase in pGL3-reporters and Renilla luciferase in pRL-TK were determined 48 h later by dual luciferase reporter assays following the manufacturer’s protocol (Promega, Madison, WI). Each transfection was performed in triplicate and each assay was repeated at least three times.

### Flow cytometric assay

To study the effects of T-5224 on the differentiation of progenitor cells to BMMCs, cells were treated with 20 µM T-5224. Culture media were changed every 3 days and differentiation was assessed every 6 days. Single cell suspensions from the BMMCs were stained with the antibody mix including CD177-PE and FcεRIα-APC (Miltenyi Biotec GmbH, Bergisch Gladbach, Germany). To set the gates, flow cytometric dot plots were based on comparison with isotype controls, fluorescence minus one, and unstained cells. The percentage of CD177 and FcεRIα double-positive cells were measured by flow cytometry (CytoFLEX, Beckman Coulter, Miami, FL).

### IgE-mediated passive cutaneous anaphylaxis (PCA) mouse model

A PCA mouse model was used to assess T-5224 effects on allergic reactions [31]. In this model, extravasation reflects vessel permeability. Twenty-four mice were randomized into four groups (6/group): PBS; DNP-HSA; DNP-HSA plus T-5224; and DNP-HSA plus ketotifen. Left ears were injected subcutaneously with anti-DNP IgE (500 ng in 0.9% saline). Twenty-four hours later, IgE-sensitized mice were given an intraperitoneal (i.p.) injection of T-5224 (60 mg/kg) or ketotifen (50 mg/kg), and 1 h later challenged with a tail vein injection of 200 µg of DNP-HSA in 1× PBS with 0.5% Evans blue dye. Mice were euthanized 1 h after the challenge; their ears were removed and dissolved in 700 μL formamide overnight at 62 °C. Extravasated dye was quantitated with 620-nm spectrophotometry (Bio-Rad, Hercules, CA).

### Ovalbumin (OVA)-induced active systemic anaphylaxis (ASA) mouse model

The OVA-induced ASA model is widely used to examine immediate-type hypersensitivity, which is strongly associated with MCs [32]. A cohort of 24 mice was randomized into four groups (6/group): negative control; OVA sensitization/challenge; OVA sensitization/challenge plus T-5224; and OVA sensitization/challenge plus ketotifen (50 mg/kg). The mice were sensitized on days 0 and 7 with OVA (100 µg i.p. with 2 mg alum adjuvant). From experimental day 9 to 13, mice received T-5224 (60 mg/kg) and ketotifen (50 mg/kg), i.p. on alternating days. On day 14, the mice were challenged with 200 μg OVA (i.p.) and then monitored for 90 min, during which their rectal temperatures were measured every 10 min. Immediately thereafter, orbital venous plexus blood was sampled for serum histamine and IL-4 level measurement by enzyme-linked immunosorbent assay (ELISA).

### Oxazolone (OXA)-induced atopic dermatitis model

In the OXA-induced atopic dermatitis model, massive inflammatory cell infiltration leads to skin thickening [33]. To produce the model [34], the right ears of Balb/c mice were sensitized with 20 μL of 1% OXA (TargetMol, Shanghai, China) in 4:1 (v/v) acetone-olive oil mixture (experimental day 0). Plain acetone/sesame seed oil was injected in the left ears to obtain within-animal controls. On days 7, 9, and 11, the ears were challenged with 20 μL of 1% OXA three times a week for 1 week. At the same time, T-5224 (60 mg/kg, i.p.) was administered. On day 13, the mice were sacrificed, ear samples were collected for further analysis. Ear swelling change was used as a measure of allergic response.

### Cytology and histology

Cell staining methods are described in Additional file 1 (Supplementary Materials and Methods 1.1). Cells were examined under a light microscope (Carl Zeiss, Goettingen, Germany) to view staining and to detect morphological changes associated with MC degranulation, particularly F-actin cytoskeleton decomposition [35]. Mouse ears were fixed in a 4% paraformaldehyde (Solarbio, Beijing, China) and embedded in paraffin. Sections were cut (thickness, 4 mm), dewaxed, cleared in xylene, hydrated, and stained with hematoxylin and eosin (H&E; Seivicebio, Wuhan, China) to visualize epidermal thickness and eosinophils, and with toluidine blue (Seivicebio, Wuhan, China). Eosinophils and MCs were counted in five randomly selected images.

### Histamine and IL4 release

BMMCs were sensitized with anti-DNP IgE (50 ng/mL) overnight, pretreated with T-5224 for 1 h, and then stimulated with DNP-HSA for 30 min (for histamine detection) or 6 h (for IL-4 detection). Cell suspensions were centrifuged (300×*g*, 5 min) to separate cells from media. Supernatant concentrations of histamine and IL4 were determined with enzyme-linked immunosorbent assay (ELISA) kits for mouse histamine (IBL, Hamburg, German) and mouse IL4 (Shanghai Hu Zhen Biological Technology, Shanghai, China).

### Statistical analyses

All clinical and histological evaluations were performed in a blinded manner. Data were analyzed in Prism 8 (GraphPad, La Jolla, CA). For normally distributed quantitative variables, means of triplicate values are reported with standard deviations (SDs). Kruskal–Wallis tests were performed to detect significant differences between treatment groups. One-way analyses of variance (ANOVAs) and Dunnett’s post‑hoc tests were used to find differences between treatment groups in the in vivo experiments. Differences was considered significant at *p* < 0.05.

## Results

### c-Fos expression is elevated in IgE-stimulated MCs

**Fig. 1:**
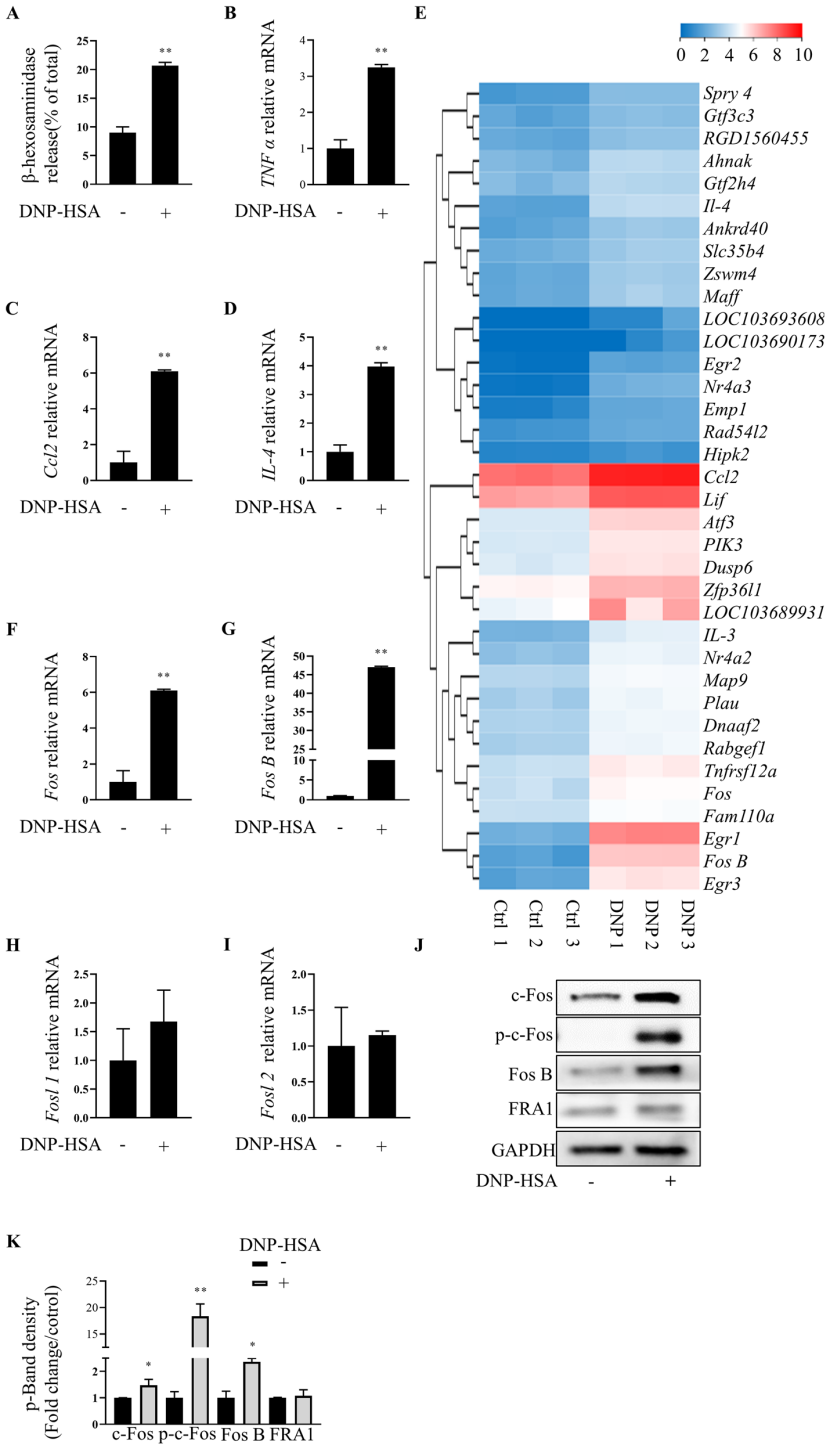
c-Fos expression is elevated in IgE-stimulated MCs. **A** IgE-sensitized RBL-2H3 were stimulated by DNP-HSA for 30 min, β-hexosaminidase released into the supernatant was measured. **B**–**D** IgE-sensitized RBL-2H3 were stimulated by DNP-HSA for 4 h, the gene expression of pro-inflammatory cytokines TNF α, Ccl2 and IL 4 was analyzed by RT-PCR. **E** Heatmap of 36 selected DEGs. **F**–**I** The gene expression of Fos family (c Fos, Fos B, Fosl1, Fosl2) was determined by RT-PCR. **J** Western blot for c-Fos family protein members (c Fos, p-c Fos, FRA 1, Fos B). **K** Band signals were analyzed by Image J software. Data summarize three independent experiments; *p < 0.05 **p < 0.01 vs. nontreated activated cell

In IgE-activated RBLs, DNP-HSA triggered the release of the granule-related mediator β-hexosaminidase (*p* < 0.01; Fig. 1A) and increased transcription of inflammatory cytokine genes associated with type Ι hypersensitivity reactions, namely *TNF*, *CCL2*, and *IL4* (all *p* < 0.01; Fig. 1B–D), affirming the establishment of a MC activation model. In non-stimulated MCs, RNA-seq revealed 191 differentially expressed genes (DEGs; Q ≤ 0.05) following DNP-HSA treatment. Among them, 116 were upregulated (61%) and 75 were down-regulated (39%; Additional file 1: Figure S1). The top 36 upregulated genes are shown in Fig. 1E. Functional enrichment analysis indicated that there were functionally distinct modules within each group (Additional file 1: Figures S2 and S3).

RT-qPCR assays demonstrated increased mRNA levels of *cFos* (Fig. 1F) and *FosB* (Fig. 1G) in stimulated RBLs (both *p* < 0.01), with unchanged *Fosl1* (Fig. 1H) and *Fosl2* (Fig. 1I) levels (both *p* > 0.05). Western blotting showed that activated MCs had significantly upregulated c-Fos and FosB after treatment with DNP-HSA (Fig. 1J), together with increased phosphorylation of c-Fos at Ser32 (Fig. 1J).

### c-Fos upregulates EGR1 transcription through EGR1 promoter binding

**Fig. 2:**
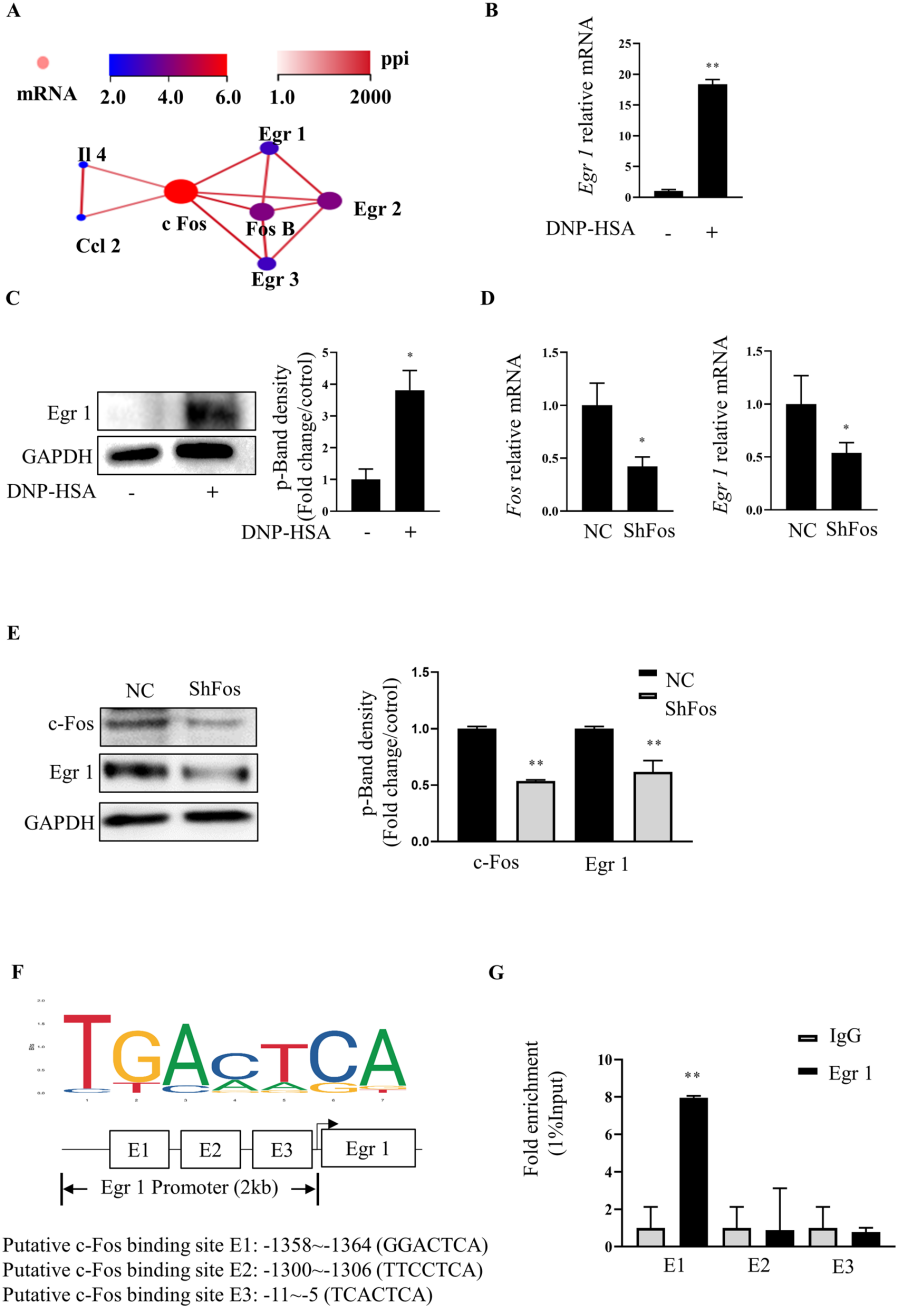
Fos up-regulates transcriptionally the EGR1 expression through binding to EGR1 promoter. **A** Protein–protein interaction (PPI) network. **B**, **C** The gene expression of pro-inflammatory cytokines Egr 1 was analyzed by RT-PCR and Western blot. **D**, **E** RT-PCR and Western blot analysis of Egr 1 expression in RBL‑2H3 cells transfected with ShRNA‑Fos. **F** c Fos binding site on the Egr 1 promoter region was predicted by consulting the JASPAR database. **G** Interaction of c Fos with Egr 1 promoter region was validated by CHip assay. *p < 0.05 **p < 0.01. Sh-NC, negative control

Network analysis suggested c-Fos protein–protein interactions with a number of MC activation-associated proteins, including early growth response 1 (Egr1), Egr2, Egr3, IL-4, and Ccl2 (Fig. 2A). Subsequently, qRT-PCR and western blot analyses showed that IgE/Ag-stimulated MCs had increased *Egr1* mRNA and EGR1 protein levels, compared to non-stimulated MCs (Fig. 2B, C), consistent with our RNA-Seq data. Treatment with shFos decreased EGR1 mRNA and protein levels, compared to non-treated controls (NCs) (Fig. 2D, E). To verify that EGR1 is activated by c-Fos in MCs, we obtained the first three Fos-binding sequences within the *Egr1* promoter from JASPAR (Fig. 2F). A ChIP assay confirmed that Fos binds the *Egr1* promoter (Fig. 2G).

### Inhibition of c-Fos/AP1 attenuates IgE-mediated MC activation in vitro

**Fig. 3:**
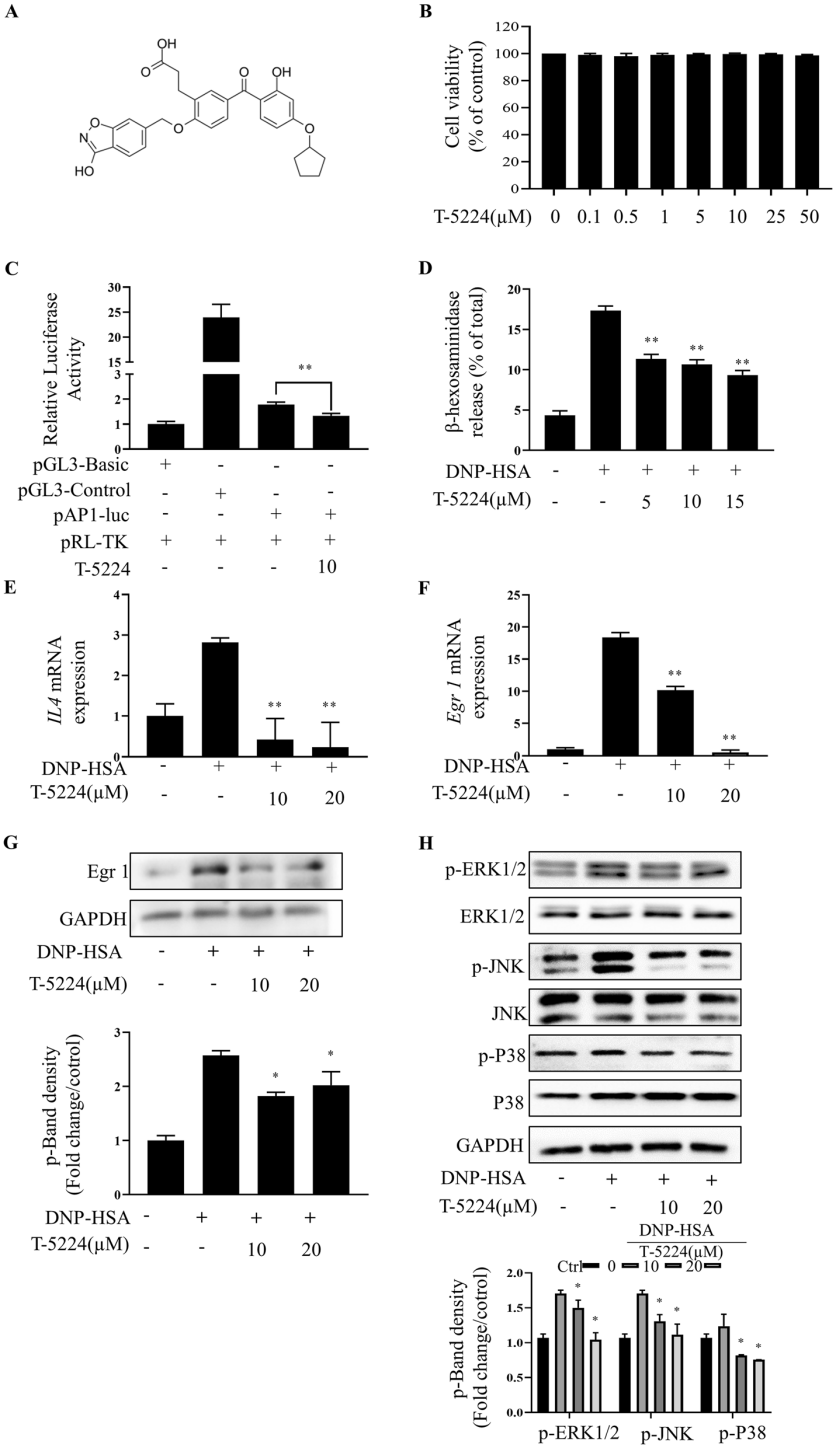
The transcription factor c-Fos/AP-1 inhibitor T-5224 shows its anti-allergic effects on IgE/Ag activated RBL-2H3 cells. **A** Chemical structure of T-5224. **B** RBL-2H3 was incubated with T-5224 (0.1–50 μM). After 24 h, cell viability was measured by performing the CCK-8 assays. **C** Dual luciferase reporter assays performed to detect the selective inhibitory effects on AP1 signaling pathway in MCs. **D** IgE-sensitized RBL-2H3 were pre-incubated for 1 h with indicated concentration of T-5224 and then stimulated with DNP-HSA for 30 min; β-Hexosaminidase released into the supernatant was measured. **E**, **F** The gene expression of pro-inflammatory cytokines IL-4 and Egr 1 was analyzed by RT-PCR. **G**, **H** Total protein was extracted, and the protein samples were analyzed by western blotting using specific antibodies (Egr 1, ERK, p-ERK, JNK, p-JNK, p38, p-p38). The band shown is representative of three independent experiments. Band signals (left panel) were analyzed by Image J software and the fold increase of signal levels compared with the Ctrl group was measured (right panel). Ctrl: control group without DNP-HSA treatment. Data summarize three independent experiments; *p < 0.05 **p < 0.01 vs. nontreated activated cell

The chemical structure of T-5224, a selective c-Fos/AP1 inhibitor, is shown in Fig. 3A. Cell viability assays showed that ≤ 50 µM T-5224 exposure for 24 h did not have cytotoxic effects on RBLs (Fig. 3B). Thus, we used ≤ 50 μM T-5224 for our in vitro experiments. Luciferase reporter (Luc) assays performed to detect off-target versus selective AP1-inhibitory effects showed that T-5224 reduced AP1-Luc activity (Fig. 3C). In DNP-HSA-activated RBLs, T-5224 reduced β-hexosaminidase release (IC_50_: 18.30 ± 1.48 µM; Fig. 3D) and suppressed IL4 release (Fig. 3E). T-5224 reduced activation-associated cytomorphological changes, including elongation and F-actin decomposition (Additional file 1: Figure S4).

Regarding AP1-inhibition effects on MC activation signaling, qRT-PCR and western blot analyses showed that T-5224 pretreatment decreased transcription and translation of EGR1 in IgE-stimulated RBLs, with the effect being stronger with the high dose (20 µM) than with the low dose (10 µM) (Fig. 3F, G). Western blot analysis showed that T-5224 inhibited activation of MAPKs (p38, JNK, and ERK) in activated RBLs, as evidenced by decreased levels of p-p38, p-JNK and p-ERK1/2 (Fig. 3H), with the high dose resulting in more pronounced reductions.

**Fig. 4:**
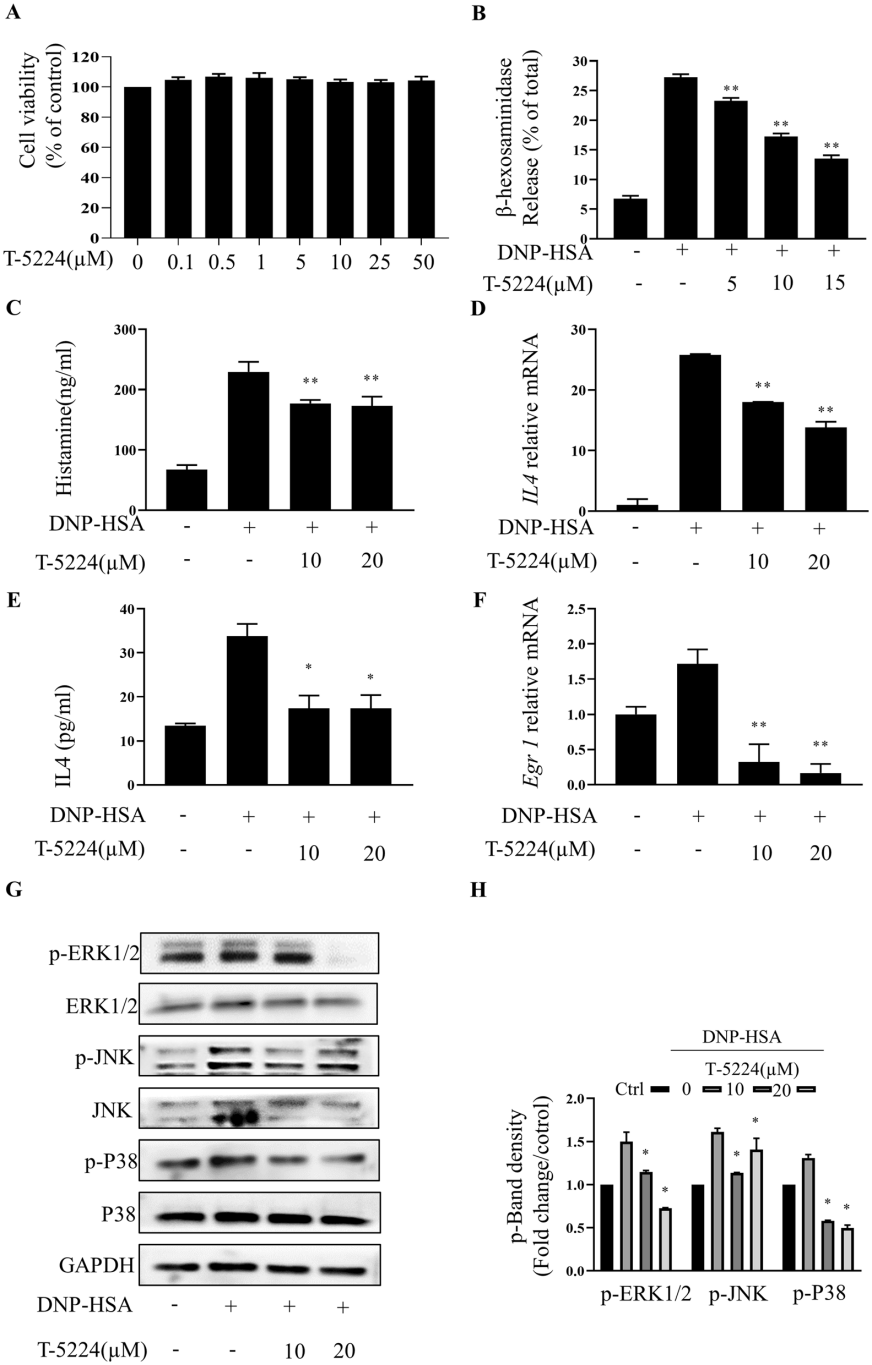
T-5224 attenuates IgE-mediated BMMCs activation. **A** RBL-2H3 was incubated with T-5224(0.1–50 μM). After 24 h, cell viability was measured by performing the CCK-8 assays. IgE-sensitized BMMCs were pre-incubated for 1 h with indicated concentration of T-5224 and then stimulated with DNP-HSA for 30 min; β-hexosaminidase (**B**) and histamine (**C**) released into the supernatant was measured. **D** The gene expression of inflammatory cytokine IL-4 was determined by RT-PCR. **E** The secretion of inflammatory cytokines IL-4 was measured by ELISA. **F** The gene expression of Egr 1 was determined by RT-PCR. **G**, **H** Total protein was extracted, and the protein samples were analyzed by western blotting using specific antibodies (Egr 1, ERK, p-ERK, JNK, p-JNK, p38, p-p38). The band shown is representative of three independent experiments. Band signals (left panel) were analyzed by Image J software and the fold increase of signal levels compared with the Ctrl group was measured (right panel). Ctrl: control group without DNP-HSA treatment. Data summarize three independent experiments; *p < 0.05 vs. nontreated activated cell

T-5224 (0.1–50 μM) did not affect BMMC viability (Fig. 4A). T-5224 inhibited release of β-hexosaminidase (IC_50_: 14.51 ± 0.44 µM; Fig. 4B) and histamine (Fig. 4C) in IgE/Ag-stimulated BMMCs. T-5224 pretreatment reduced *IL4* mRNA, IL4 secretion, and *Egr1* mRNA expression with the high-dose treatment producing more pronounced effects than the low-dose treatment (Fig. 4E–G). Concomitantly, T-5224 pretreatment inhibited activation of all three MAPK pathways (Fig. 4F).

### T-5224 inhibits IgE-mediated PCA in vivo

**Fig. 5:**
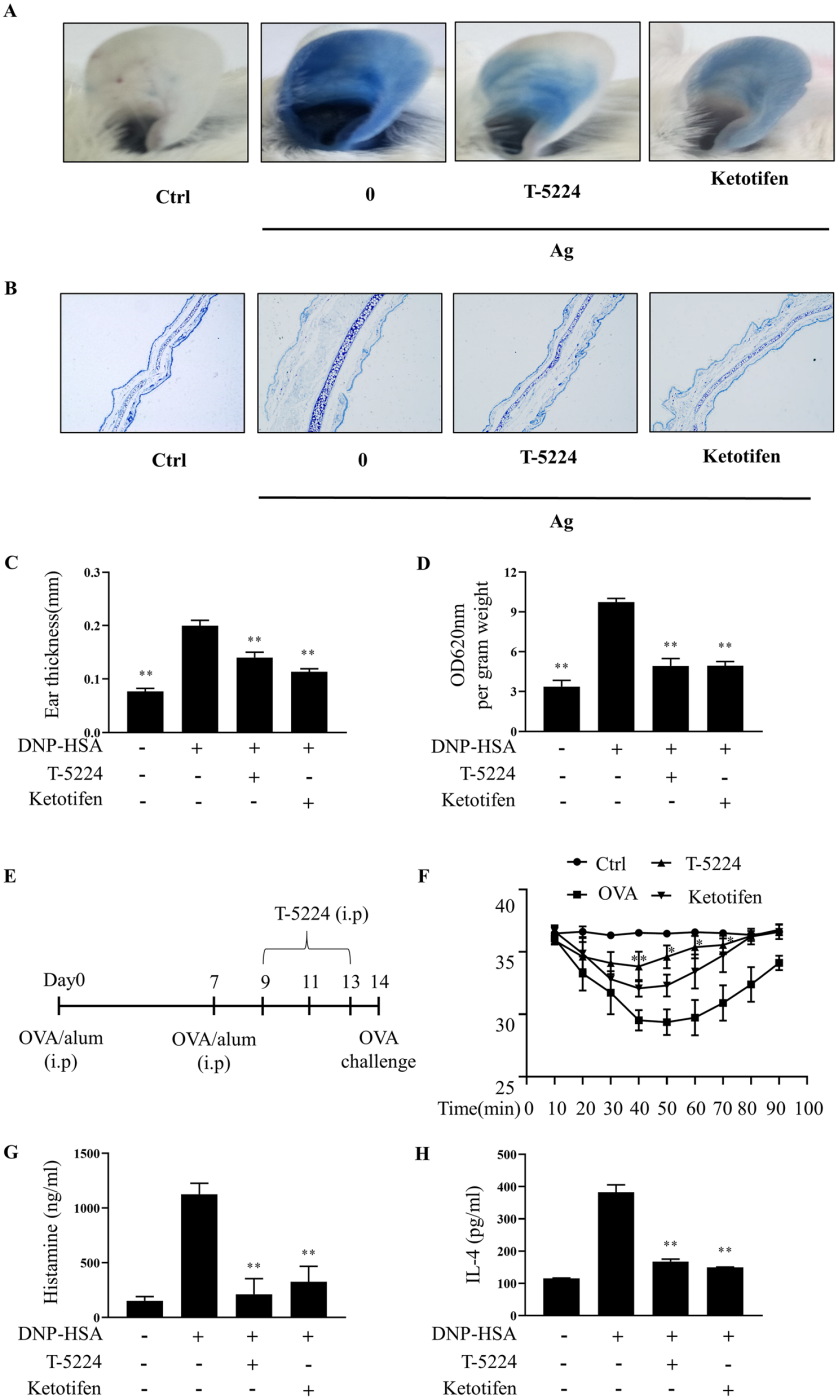
T-5224 inhibits IgE-mediated allergic response in PCA and ASA model. In a PCA test, Balb/c mice were injected intradermally with anti-DNP IgE for 24 h and with intravenous DNP-HSA containing Evans blue. **A**, **B** The representative images of ears and photomicrographs of ear sections were stained with toluidine blue as shown. **C** Ear thickness was measured. **D** The dye extracted from the ear was detected using a spectrophotometer. **E** ASA model protocol (n = 6/group). Ketotifen was used as a positive control treatment. **F** Rectal temperatures were measured every 10 min for 1.5 h. Blood was obtained from the abdominal vein of each mouse to measure serum histamine, IL-4. Serum histamine (**G**) and IL-4 levels (**H**) were determined by ELISA. Means ± SDs of 3 independent experiments are shown; *p < 0.05 vs. non-treated mice

As shown in Fig. 5, IgE/Ag stimulation induced robust in vivo PCA reactions, evidenced by dye diffusion, ear thickening, and dye extrusion from injected ears. T-5224 attenuated these responses, as shown by decreased dye diffusion (Fig. 5A, B), reduced ear thickness (Fig. 5C), and diminished dye extrusion (Fig. 5D) compared to the activated group not given T-5224. The effects of T-5224 resembled those of the anti-allergy drug ketotifen.

### T-5224 attenuates MC-induced allergic reaction in OVA-induced ASA model

To establish the OVA-induced allergy model (Fig. 5E), mice were sensitized with repeat administrations of OVA Ag plus alum adjuvant; then an allergic reaction was induced with an OVA challenge (Fig. 5E). During the 90-min post-OVA challenge period, rectal temperatures of control mice decreased over 40–50 min, and then recovered. This reduction was attenuated by injection of T-5224 or ketotifen (positive control) (Fig. 5F). Notably, 80 min after the challenge induction, the mean rectal temperature for the OVA-sensitized group remained low (29.36 ± 0.93 °C) whereas that of the T5224-treated OVA-sensitized group (34.60 ± 0.83 °C) was similar to that of non-sensitized controls. Concomitantly, serum histamine levels increased after the OVA challenge (non-sensitized control group, 1126 ± 81 ng/mL) and this increase was diminished significantly by T-5224 (211 ± 122 ng/mL) (Fig. 5G). The similar result was found in the serum IL4 levels (Fig. 5H).

### T-5224 suppresses OXA-induced atopic dermatitis symptoms in mice

**Fig. 6:**
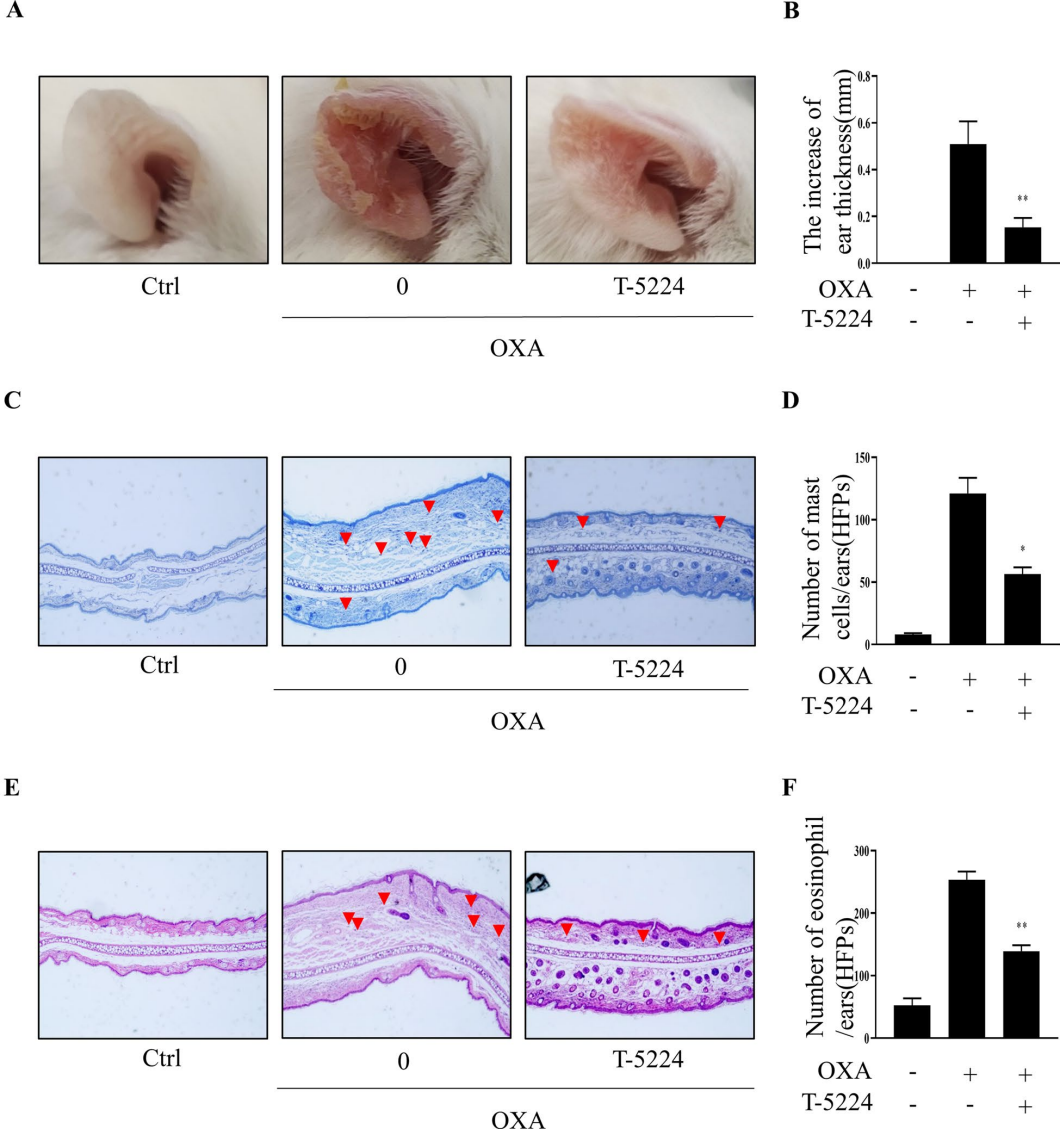
T-5224 suppresses MCs activation mediated AD induced by OXA in mice. **A** Clinical features of AD-like skin lesions treated with T-5224. **B** T-5224 attenuates ear swelling in OXA-induced AD-like ear lesions. **C** Mast cell infiltration (red arrows) stained with toluidine blue in ear, **D** mast cells measured under 10–10 high-power fields (HPFs) in ear. **E** Eosinophil infiltration (red arrows) in ear, determined by hematoxylin and eosin (H&E) stain, **F** the number of eosinophils infiltrating the ear skin measured under 10–10 high-power fields (HPFs). *p < 0.05, **p < 0.01 vs. nontreated mice

The atopic dermatitis model mice exhibited marked ear skin thickening and these dermatitis symptoms were attenuated by T-5224 (*p* < 0.01; Fig. 6A, B). Toluidine blue staining showed that the infiltration of mast cells in the dermis was observed in the dermis of the control group. The number of mast cells in the ear dermis was decreased significantly after T-5224 treatment (*p* < 0.01; Fig. 6C, D). In addition, increased the permeability of blood vessels in allergic skin allowed eosinophils to infiltrate into tissue. H&E staining revealed that eosinophil infiltration of the ear skin was attenuated by treatment in the T-5224 groups compared with the AD group (*p* < 0.01; Fig. 6E, F). The results indicated T-5224 had a protective effect against OXA‑induced AD in mice.

## Discussion

We confirmed that DNP-HSA triggered MC degranulation, as evidenced by β-hexosaminidase release and upregulation of allergic reaction-related genes, thereby confirming the validity of our IgE-activated RBL model of activated MCs [36]. Transcriptome analysis revealed that *cFos* and *FosB* were upregulated by DNP-HSA stimulation of RBLs. Stimulated RBLs had increased c-Fos phosphorylation. Inhibition of c-Fos/AP1 with T-5224 reduced degranulation, suppressed MC activation-associated morphological changes, and attenuated MC activation effects on IL4 and EGR1 expression. IgE/Ag-stimulated MCs showed upregulation of EGF1, both transcriptionally and translationally, that could be attenuated by suppression of c-Fos activity. We demonstrated in a ChIP assay that Fos bound the *Egr1* promoter region, indicating that *Egr1* transcription was likely activated by c-Fos binding its promoter during MC activation. Furthermore, T-5224 pretreatment decreased EGF1 transcription and translation and inhibited MAPK activation in activated MCs. Finally, in multiple models, T-5224 had a protective effect against the development of allergic responses including swelling, body temperature reduction, histamine release, and infiltration of MCs and eosinophils.

MCs represent a potential therapy target to alleviate allergic reactions [37]. Extending prior findings implicating *Fos* upregulation in IgE/FcεRI-activated MCs [13], we demonstrated that IgE/Ag treatment enhances c-Fos transcription and translation, and that c-Fos acts on the *Egr1* promoter to upregulate EGR1 expression. EGR1 is a zinc-finger transcription factor that regulates the expression of various inflammatory cytokines following FcεRI activation, including TNF, IL6, IL13, and IL4 [38, 39].

Previously, EGR1 was shown to induce IL4 secretion in FcεRI-activated MCs by binding the *Il4* promoter [21]. IL4 enhances IgE-mediated MC responses and is a necessary mediator of allergy development [20, 40]. IL4 is critical for directing Th2 cell differentiation and B cell antibody class switching to allergenic IgEs [41]. FcεRI-mediated MC-derived IL4 production is important for allergy pathogenesis [42]. Our results indicating that c-Fos binding of the *Egr1* promoter can drive EGR1 expression are consistent with a new cFos/Egr1/IL-4 axis of MC activation.

AP1 activity is positively associated with the development of IgE-mediated allergic diseases, and AP1 inhibition can suppress allergic asthma symptoms [43–46]. To examine the allergenic role of AP1, we employed T-5224, a selective c-Fos/AP1 inhibitor produced by Aikawa et al. based on the crystal structure of the AP1-DNA complex [47]. T-5224 has been shown to suppress collagen-induced arthritis [47], lipopolysaccharide-induced liver/kidney injury [48, 49], intervertebral disc degeneration and associated pain [50], and breast cancer [51]. However, it was not known whether T-5224 may impede MC-induced allergic responses.

Our results showing that T-5224 reduced FcεRI-mediated degranulation in MCs were similar to previously reported effects of palbociclib (cyclin-dependent kinase inhibitor) and tozasertib (Aurora kinase inhibitor) [22, 52]. Mechanistically, T-5224 decreased IgE/Ag-induced expression of EGR1 and IL4 and also blocked MAPK activation in activated MCs. A variety of established medicines, including omeprazole (proton pump inhibitor) [7], tozasertib [52], and berberine (adenosine monophosphate-activated protein kinase stimulator) [53] inhibit MC activation-associated MAPK signaling. Meanwhile, T-5224 did not affect maturation of precursor cells into MCs (Additional file 1: Figure S5). Together, these data support exploration of T-5224 for potential repurposing as a MC activation inhibitor. We proceeded to conduct in vivo studies in murine models of allergic disease employing doses appropriate for clinical application (30–300 mg/kg).

Previously, oral administration of T-5224 (30 mg/kg per day for 3 weeks) was shown to resolve collagen-induced arthritis in a preclinical model [47]. Additionally, oral administration of T-5224 (300 mg/kg) has been shown to ameliorate lipopolysaccharide-induced liver/kidney injury in mice through a mechanism that involves reducing production of proinflammatory cytokines [48, 49]. T-5224 was developed into a potential therapeutic agent for rheumatoid arthritis and has advanced into phase-II clinical trials [54]. The major metabolites of T-5224, glucuronides, occur predominantly in the liver [55]. The T-5224 treatments employed in our PCA and ASA model mice, which translate to a 60-mg/kg dose, showed robust effectiveness without signs of cytotoxicity. We proceeded to test T-5224’s ability to suppress MC activation in a model of atopic dermatitis, which is characterized by MC activation and intra-dermal infiltration of MCs that release vasoactive and proinflammatory mediators [56, 57]. T-5224 attenuated MC and eosinophil infiltration in OXA‑induced allergic skin, consistent with a protective effect against allergic dermatitis.

In conclusion, the present experiments demonstrated a previously unknown molecular mechanism whereby IgE/Ag promotes MC activation via a c-Fos/Egr1/IL4 axis. The c-Fos/AP1 inhibitor T-5224 reduced MC degranulation and cytokine production and attenuated allergic responses in vivo. The ability of T-5224 to inhibit MC degranulation and allergic disease presentation suggest that it should be considered as a candidate for repurposing into an allergic/inflammatory disease pharmacotherapy. Clinical trials aimed at determining the anti-allergy efficacy and toxicity of T-5224 are warranted.

## Supplementary Information

**Additional file 1.** **Supplementary Materials and methods 1.1**. Toluidine blue and F-actin staining. **Supplementary Materials and methods 1.2**. MC differentiation assay. **Figure S1**. Volcano plot of RNA-seq data. **Figure S2**. Results of KEGG pathway analysis of upregulated DEGs. **Figure S3**. Results of GO enrichment analysis of upregulated DEGs. **Figure S4**. T-5224 reduces morphological changes in stimulated MCs. **Figure S5**. Non-effect of T-5224 on maturation of precursor cells to MCs. **Table S1**. Primers sequences for quantitative real-time PCR.

## Data Availability

The datasets used and/or analysed during the current study are available from the corresponding author on reasonable request.

## Abbreviations

AD: Atopic dermatitis
Ag: Antigen
AP-1: Activator protein 1
ASA: Active systemic anaphylaxis
BMMC: Bone marrow-derived mast cell
DNP: Dinitrophenol
Egr 1: Early growth response factor 1
ERK1/2: Extracellular signal-regulated kinase 1/2
FITC: Fluorescein isothiocyante
FcεRI: High-affinity IgE Fc receptor
GAPDH: Glyceraldehyde 3-phosphate dehydrogenase
HSA: Human serum albumin
Ig: Immunoglobulin
JNK: C-Jun N-terminal kinase
MAPK: Mitogen-activated protein kinase
MC: Mast cell
OVA: Ovalbumin
OXA: Oxazolone
PCA: Passive cutaneous anaphylaxis
