# Supplementary material for: Inhibition of c-Fos expression attenuates IgE-mediated mast cell activation and allergic inflammation by counteracting an inhibitory AP1/Egr1/IL-4 axis
Source: J Transl Med. 2021 Jun 15;19:261. doi: 10.1186/s12967-021-02932-0 (PMC8207675; doi:10.1186/s12967-021-02932-0)

**Additional file 1**

**Supplemental information**

Inhibition of c-Fos expression attenuates IgE-mediated mast cell activation and allergic inflammation by counteracting an inhibitory AP1/Egr1/IL-4 axis

Hui-Na Wang^†^, Kunmei Ji^†^, Li-Na Zhang, Chu-Chu Xie, Wei-Yong Li, Zhen-Fu Zhao, Jia-Jie Chen^*^

Department of Biochemistry and Molecular Biology of Health Science Center, Laboratory Department of Pinghu Hospital, Shenzhen University, Shenzhen 518060, China.

^†^ These authors have contributed equally to this work.

*Correspondence to: Jia-Jie Chen, E-mail address: [chenjj@szu.edu.cn](mailto:chenjj@szu.edu.cn); Department of Biochemistry and Molecular Biology of Health Science Center, Laboratory Department of Pinghu Hospital, Shenzhen University, No. 1066 Xueyuan Road, Nanshan District, Shenzhen, 518060, PR China; Tel: +86-755-86671979; Fax: +86-755-86671906.

**Inventory of Supplementary information:**

Supplementary Materials and methods

Supplementary Figures S1-S5

Supplementary Tables S1

1. **Supplementary Materials and methods**

*1.1 Toluidine blue and F-actin staining*

Anti-DNP IgE (50 ng/ml)-sensitized RBLs (1.2 × 10^5^ cells/well in 24-well plates) were treated and incubated as for the β-hexosaminidase release assay. They were stimulated with DNP-HSA for 30 min and fixed with 4% paraformaldehyde in PBS for 30 min at room temperature. For toluidine blue staining, fixed cells were stained with 300 μl of toluidine blue dye (1% w/v in 0.9% saline, pH 2.5) for 30 min. For F-actin staining, fixed cells were washed with PBS and then permeated with 0.1% Triton-X100 PBS for 3 min. Stained cells were observed with inverted microscope (Carl Zeiss, Goettingen, Germany).

*1.2 MC differentiation assay*

Cells were treated with 20 µM T-5224. Culture media were changed every 3 d and differentiation was assessed every 6 d. The percentage of c-Kit and FcεRIa double-positive cells were measured by flow cytometry (CytoFLEX, Beckman Coulter, Miami, FL) using the fluoresce-labelled antibodies CD177-PE and FcεRIα-APC (Miltenyi Biotec GmbH, Bergisch Gladbach, Germany).

1. **Supplementary figure legends**

**Figure S1*. Volcano plot of RNA-seq data.*** Scatterplot of gene expression levels measured by RNA-seq analysis in non-stimulated RBLs that were treated, or not, with DNP-HSA for 4 h. DEGs (Q ≤ 0.05) are shown with red (upregulated, n = 16) and blue (down-regulated, n = 75) dots.

**Figure S2. *Results of KEGG pathway analysis of upregulated DEGs.*** Scatterplot of gene expression levels measured by RNA-seq in non-stimulated RBLs treated (or not) with DNP-HSA for 4 h.

**Figure S3. *Results of GO enrichment analysis of upregulated DEGs.*** Scatterplot of gene expression levels measured by RNA-seq in non-stimulated RBLs treated (or not) with DNP-HSA for 4 h.

**Figure S4. T-5224 reduces morphological changes in stimulated MCs.** Anti‑DNP IgE‑sensitized RBLs were pretreated (or not) with T-5224 for 1 h and then challenged with DNP‑HSA (50 ng/ml) for 30 min. (A) Representative images of RBLs stained with toluidine blue. (B) Statistical data from three independent experiments. (C) Representative images of RBLs stained with FITC-phalloidin. (D) Statistical data from three independent experiments. **p* < 0.05 ***p* < 0.01 vs. nontreated activated RBLs.

**Figure S5. Non-effect of T-5224 on maturation of precursor cells to MCs.**

(A) Scatter diagrams of MC flow cytometry analysis. Murine myeloid precursor cells from bone marrow were cultured for 6 wks with T-5224 in the presence of mIL-3 and mSCF. Cells were labelled with anti-c-KIT and anti-FcεRIα. Flow cytometric analysis was performed every 6 d. (B) Effects of T-5224 on percentages of c-KIT+ FcεRIα+ MCs. Three repeated mouse experiments with similar results were carried out; **p* < 0.05, ***p* < 0.01 vs. untreated group, one-way ANOVA.

1. **Table S1. Primers sequences for quantitative real-time PCR**

**Table S1. Primers sequences for quantitative real-time PCR**

| \| Genes \| Forward(5′-3′) \| Reverse (5′-3′) \| \| --- \| --- \| --- \| |
| --- | --- | --- | --- |
| \| *Rat GAPDH* \| GGCACAGTCAAGGCTGAGAATG \| ATGGTGGTGAAGACGCCAGTA \| \| --- \| --- \| --- \| |
| \| *Rat TNFα* \| CCCTGTTCTGCTTTCTCA \| GTTCTCCGTGGTGTTCCT \| \| --- \| --- \| --- \| |
| \| *Rat Ccl2* \| GCAGAGACACAGACAGAGG \| CCAGAAGCGTGACAGAGA \| \| --- \| --- \| --- \| |
| \| *Rat Il4* \| GAGGACCAGAACGAGACA \| CCAGAAGCGTGACAGAGA \| \| --- \| --- \| --- \| |
| \| *Rat Fos* \| CGGTCAAGAACATTAGCAACAT \| GAACCAGACAGGTCCACATC \| \| --- \| --- \| --- \| |
| \| *Rat Fos B* \| CACTTCCTCGTTTGTCCTCAC \| TCTTCCTCCTCCTCGTTCCT \| \| --- \| --- \| --- \| |
| \| *Rat Fosl 1* \| CGGAGGTTCATCTGGAGAGG \| GCTGCGGTTCTGACTCACT \| \| --- \| --- \| --- \| |
| \| *Rat Fosl 2* \| CAGTGATTACCTCCATGTCCAA \| CGACGCTTCTCCTCCTCTT \| \| --- \| --- \| --- \| |
| \| *Rat Egr1* \| CGCTGGTGGAGACAAGTTATC \| GCTGAGGATGAAGAGGTTGGA \| \| --- \| --- \| --- \| |
| \| *Mouse GAPDH* \| AAGAAGGTGGTGAAGCAGG \| GAAGGTGGAAGAGTGGGAGT \| \| --- \| --- \| --- \| |
| \| *Mouse Il 4* \| GCTAGTTGTCATCCTGCTCTTC \| GGTGTTCTTCGTTGCTGTGA \| \| --- \| --- \| --- \| |
| *Mouse Egr1*  CAGGAGTGATGAACGCAAGAG GAGCCAGGAGAGGAGTAGGA |
| \| *Chip Rat Egr1 E1* \| CCAACCATCACAAGAACCA \| TTGCGGGTGTTAGAGTCT \| \| --- \| --- \| --- \| |
| \| *Chip Rat Egr1 E2* \| GAGTGTGCCCTCAGTAGCT \| AATTGCATCTCGGCCTTGG \| \| --- \| --- \| --- \| |
| \| *Chip Rat Egr1 E3* \| AGCCCAGGATGACGGCTGTA \| CCAGGCGCGTAGAGCTGAGC \| \| --- \| --- \| --- \| |


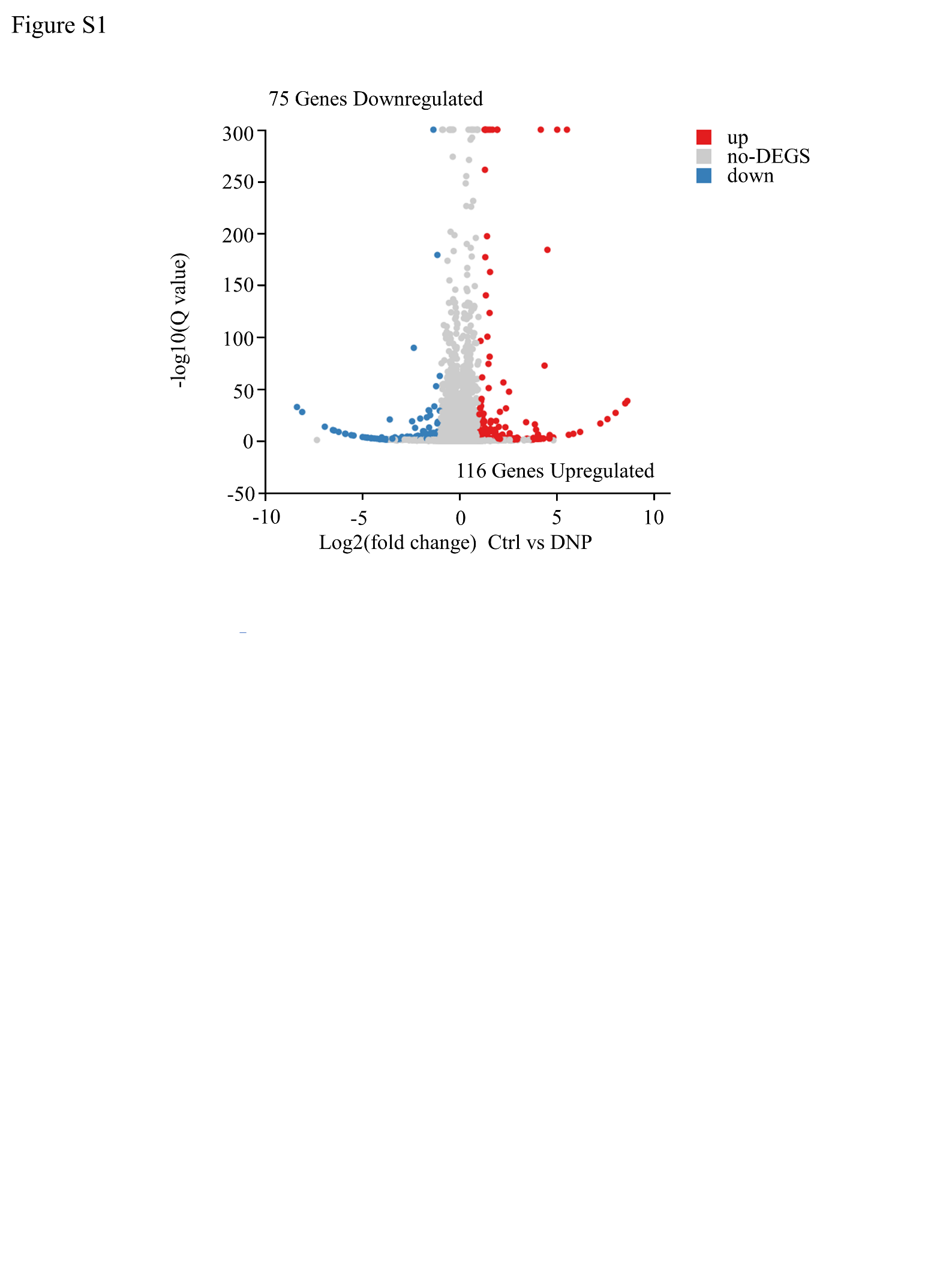


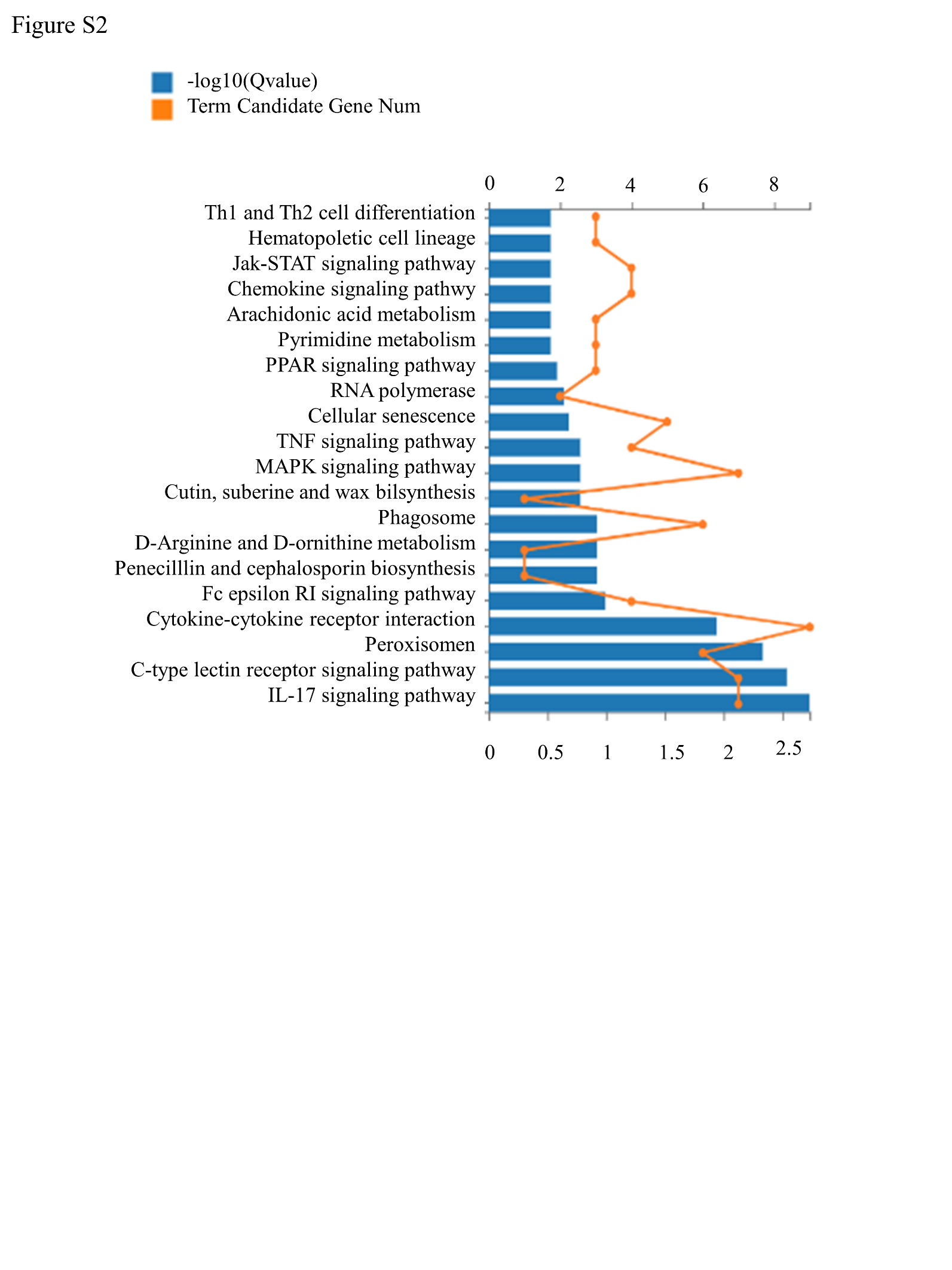


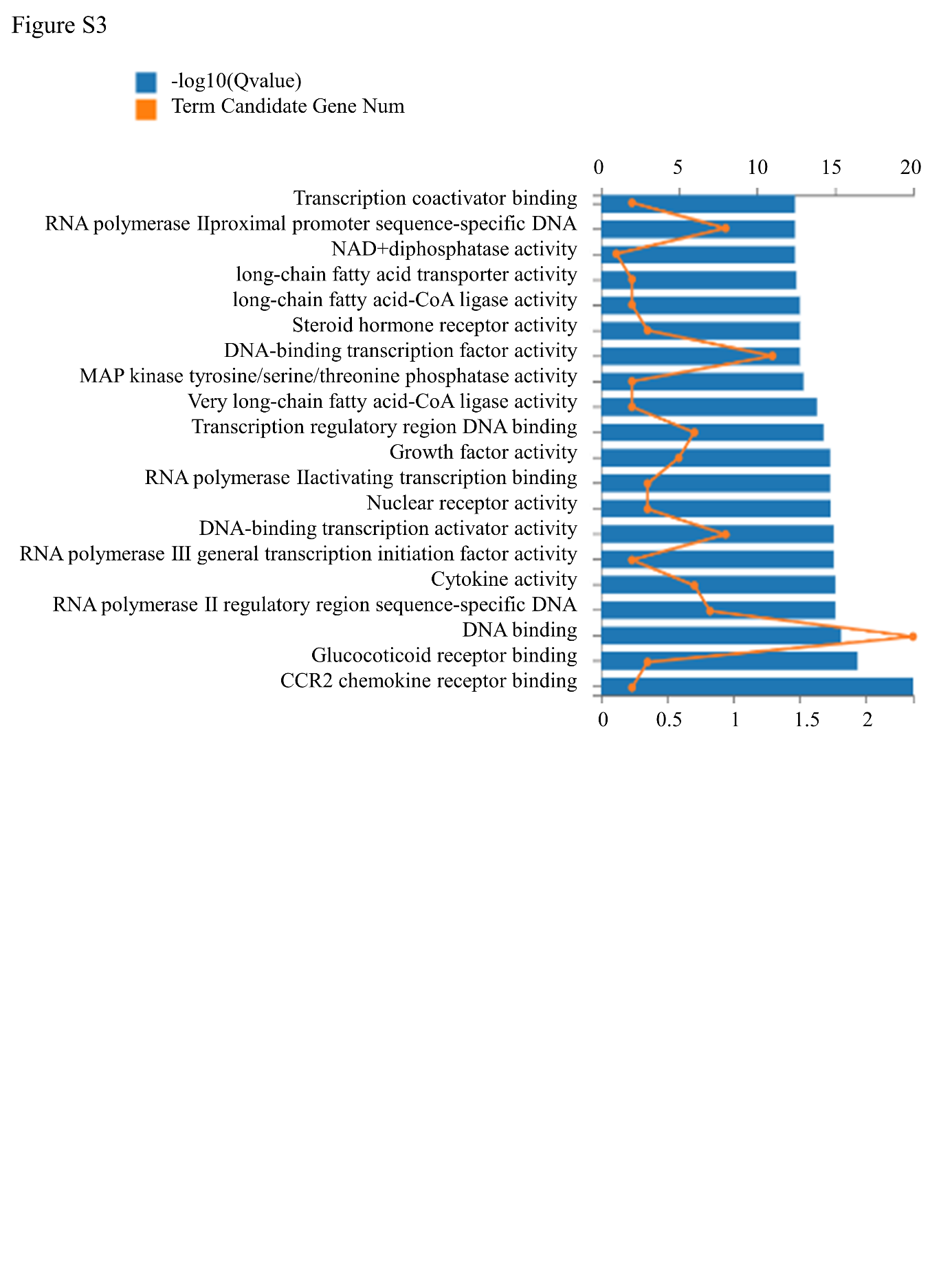


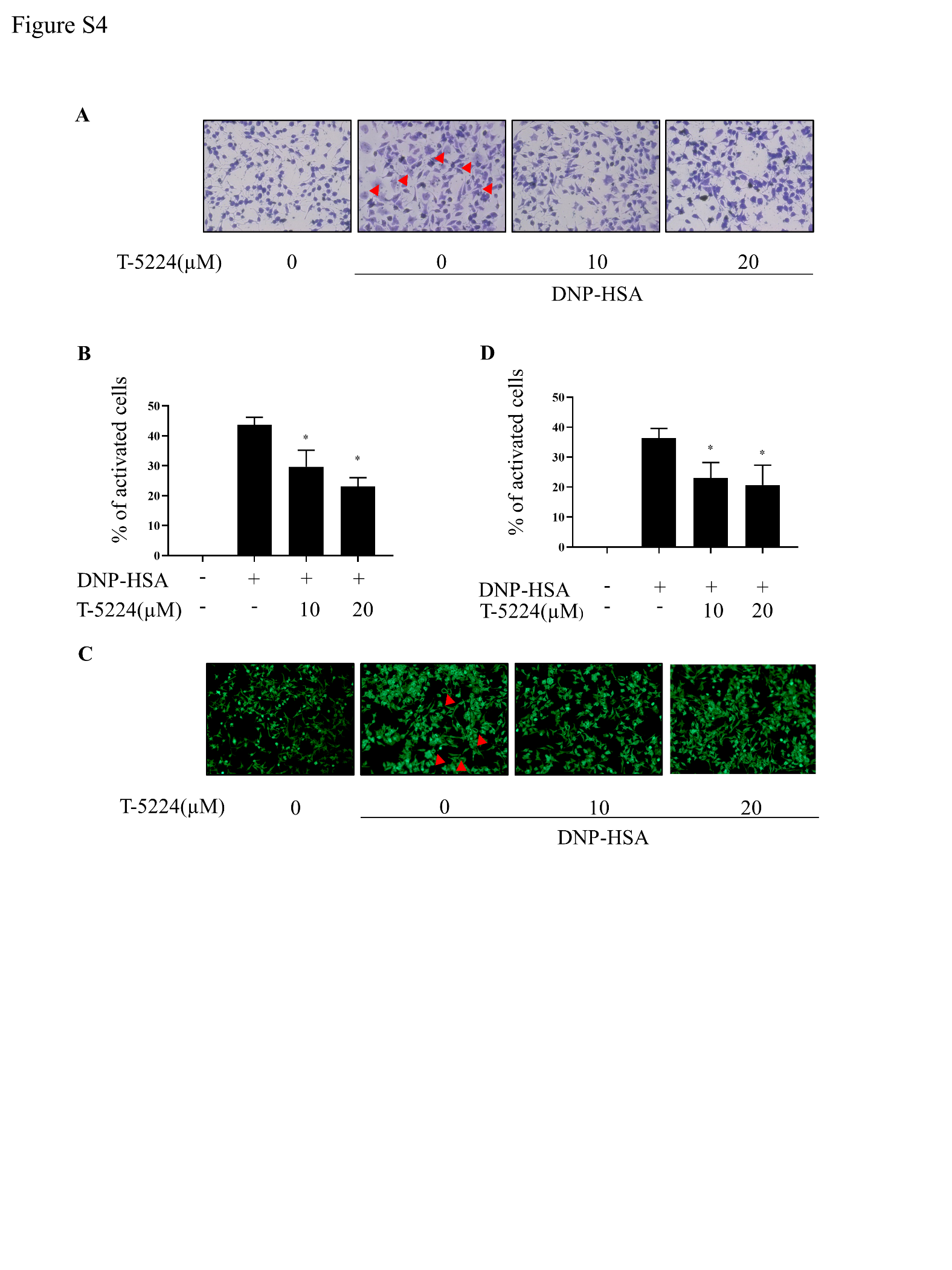


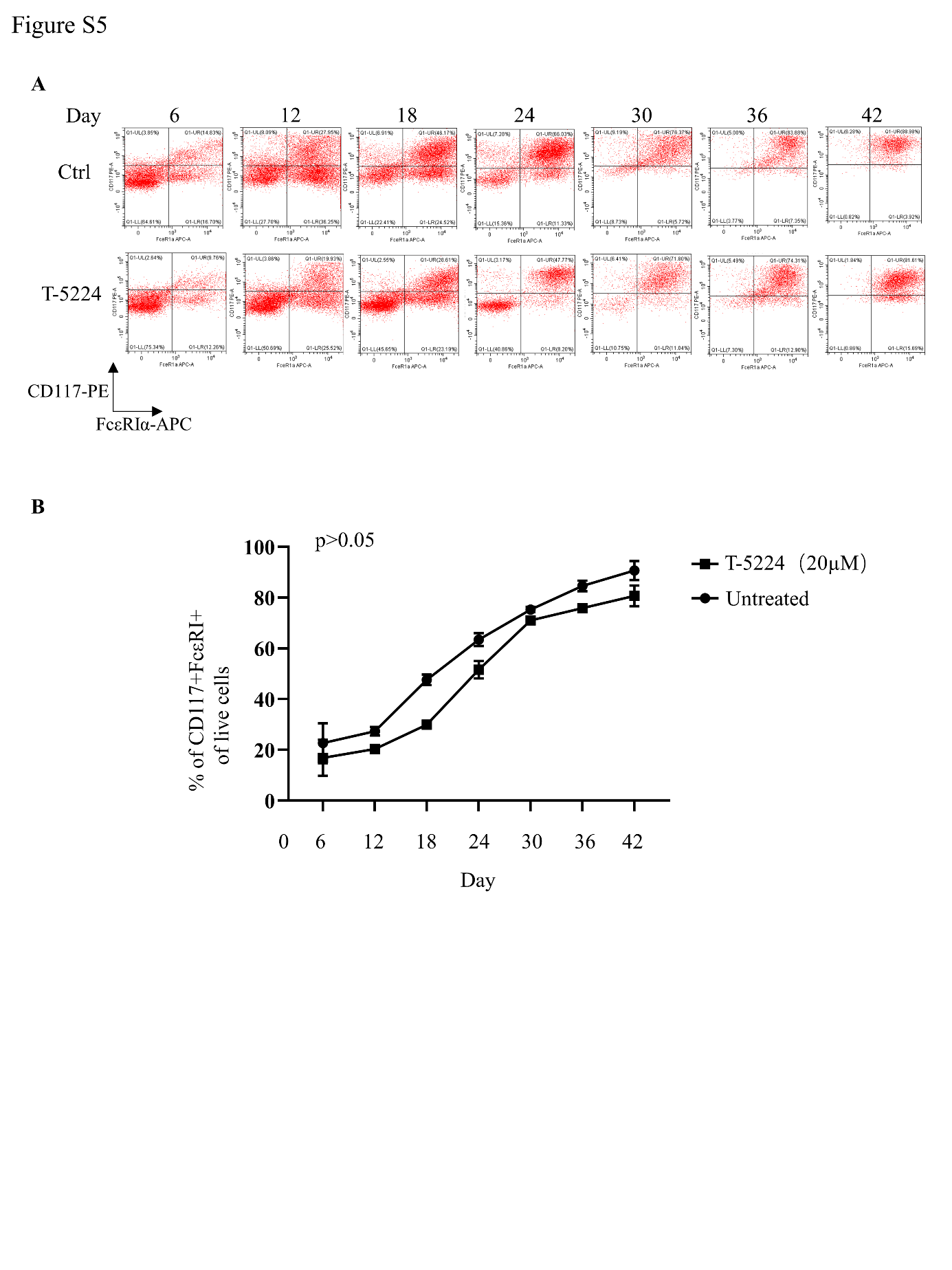

Supplement: Supplementary file 1 — Additional file 1. Supplementary Materials and methods 1.1. Toluidine blue and F-actin staining. Supplementary Materials and methods 1.2. MC differentiation assay. Figure S1. Volcano plot of RNA-seq data. Figure S2. Results of KEGG pathway analysis of upregulated DEGs. Figure S3. Results of GO enrichment analysis of upregulated DEGs. Figure S4. T-5224 reduces morphological changes in stimulated MCs. Figure S5. Non-effect of T-5224 on maturation of precursor cells to MCs. Table S1. Primers sequences for quantitative real-time PCR. [file 12967_2021_2932_MOESM1_ESM.docx]
